# Food Intelligent Quality and Safety Analysis: From Data-Driven to Data–Mechanism Hybrid-Driven Paradigm

**DOI:** 10.3390/foods15173155

**Published:** 2026-09-05

**Authors:** Zheng-Yong Zhang, Rui Zhang, Wen-Qi Yan, Min Sha

**Affiliations:** 1 School of Management Science and Engineering, Nanjing University of Finance and Economics, Nanjing 210023, China; 1120241203@stu.nufe.edu.cn (R.Z.); 1120251154@stu.nufe.edu.cn (W.-Q.Y.); minsha@nufe.edu.cn (M.S.); 2 Humanities and Social Sciences Laboratory of Jiangsu Province—Food Safety and National Strategic Governance, Jiangnan University, Wuxi 214122, China

**Keywords:** food quality, food safety, data–mechanism hybrid, intelligent analysis

## Abstract

In recent years, numerous studies have reported on intelligent analytical applications for food quality and safety. To identify the underlying patterns and development trends in this field, this paper presents a comprehensive review from the perspectives of both data-driven and mechanism-driven paradigms. Under the data-driven paradigm, detection modalities may involve either single-modal or multimodal approaches. By integrating measured detection data with appropriate intelligent learning algorithms, specific tasks for food quality or safety assessment can be achieved. Research efforts in this area encompass the development of detection techniques, optimization of measurement parameters, construction of high-dimensional spectral features, design of feature extraction methods, selection and tuning of algorithms, and formulation of multimodal data fusion strategies. This paradigm is characterized by high computational speed and superior prediction or classification efficiency. Nevertheless, it is constrained by several limitations, including poor model interpretability, limited extrapolation and generalization capabilities, and a heavy reliance on high-quality annotated data. In contrast, the data–mechanism hybrid-driven paradigm integrates physical laws and other prior knowledge as constraints that are deeply embedded into neural network training. By combining data-driven mining capabilities with theoretical prior knowledge, this approach achieves improved predictive performance and decision-making reliability. This paradigm offers notable advantages, such as enhanced interpretability, greater trustworthiness, improved data efficiency, and reduced computational costs. It is particularly well-suited for small-sample or data-sparse scenarios, and thus represents a promising and important direction for future research in this domain.

## 1. Introduction

The rapid advancement of artificial intelligence (AI) has significantly accelerated the digital transformation of food systems. For example, by employing appropriate AI models, systematic research and development of food formulations can be achieved, thereby improving research and development (R&D) efficiency [1]. AI-enhanced technologies have improved the efficiency of product quality and safety inspection, driving the digital and intelligent transformation of agriculture and food engineering [2]. AI has demonstrated considerable utility in the detection of physical, chemical, and microbiological contaminants, the assurance of quality control, and the prediction of disease outbreaks [3]. It is evident that quality and safety have consistently been important research hotspots in the field of food science. In recent years, a growing number of reports on intelligent processing methods have emerged, making a systematic review highly necessary [4]. This paper, drawing on the latest research advances, attempts to provide a comprehensive review from the novel perspective of data and mechanisms.

Food safety assessment typically targets hazardous substances including heavy metals, pesticide residues, and illegal additives. The development of intelligent technologies often involves predictive algorithms. By combining detection data with predictive algorithms, regression calculations are performed to determine the content of hazardous substances, which are then compared with the regulatory limits for food to judge whether a product meets safety requirements [5]. For instance, Mishra et al. constructed an AI-assisted antibody-functionalized electrochemical sensing platform capable of detecting and classifying Aflatoxin M1 and Atrazine in corn, corn flour, and protein matrices, achieving satisfactory performance [6]. Food quality analysis, in contrast, emphasizes stability and consistency, meaning that food quality indicators must conform to certain statistical rules of quality control [7]. The development of intelligent technologies in this domain often involves classification algorithms. By combining detection data with classification algorithms, it is possible to determine whether a food product remains within controllable categories and whether there are significant quality variations or fluctuations [8]. For example, Manini et al. developed a data-driven metal-oxide semiconductor (MOX) chemical sensing approach. By integrating sensor data with various machine learning algorithms, they successfully distinguished commercial lager beers with different alcohol contents and brands [9].

**Figure 1:**
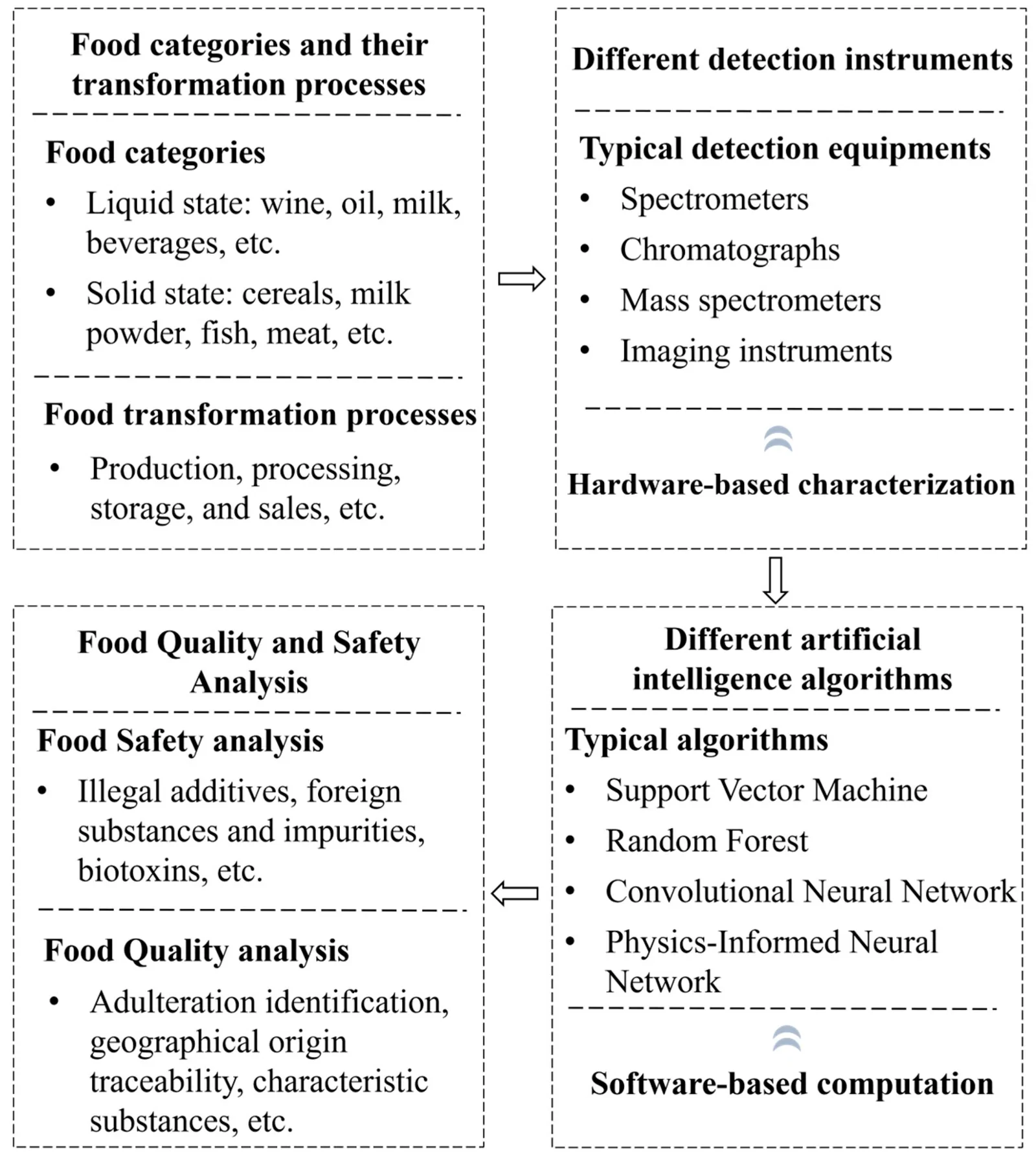
Schematic diagram of the research paradigm for food quality and safety integrating data and algorithms.

In the process of intelligent analysis for food quality and safety, existing studies vary considerably in terms of research objects, algorithms, and processing procedures [10,11]. For example, Song et al., addressing the demand for rapid, non-destructive, and data-driven monitoring in dairy product quality assessment, summarized the implementation pathways that combine various sensor technologies with intelligent algorithms, with a particular focus on research progress in feature attribution, decision explanation, and uncertainty quantification [12]. Thirumdas focused on the application of AI-related digital technologies in achieving food safety, pointing out that the integration of digital technologies with advanced detection methods holds great potential for enhancing hazard identification and critical control points in food safety management systems [13]. However, from the perspective of data, they share a commonality—almost all of them involve the efficient integration of domain-specific detection data from food samples with appropriate machine learning or deep learning algorithms, as illustrated in Figure 1. Food products encompass a wide variety of types, spanning multiple forms from raw materials to finished products, as well as multiple stages including processing and transportation. Hardware-based characterization relies on diverse analytical techniques such as spectroscopy, chromatography, and mass spectrometry, while software-based analysis ranges from conventional standard-curve quantitative methods to recent machine learning algorithms including support vector machine (SVM), random forest (RF), and convolutional neural network (CNN). These analytical targets can be further categorized into safety and quality dimensions. Unlike existing reviews that have focused predominantly on specific detection technologies (e.g., surface-enhanced Raman spectroscopy (SERS) [14], molecular imprinting [15], and novel sensors [16]), particular food categories [12], or AI-based efficiency improvements [13,17], the present review adopts a data-centric perspective that examines the entire pipeline of food characterization—from data acquisition and preprocessing to modeling and interpretation. By adopting a data-driven intelligent analysis perspective, this review aims to thoroughly investigate the data-driven analytical paradigm in the food domain, covering both single-modal and multimodal data processing pipelines, with special emphasis on the emerging trend of data–mechanism hybrid modeling. Our findings indicate that within the data-supported research paradigm, data quality is critical. Current bottlenecks include skewed data distributions, small sample sizes, and limited interpretability. Given that food materials are governed by inherent physical mechanisms, the integration of data-driven and mechanism-based hybrid analysis is increasingly emerging as a key developmental trend in intelligent food quality and safety assessment.

This review is based on the research team’s accumulated experience over many years of relevant studies. All references were retrieved from the Web of Science database, with the search focusing on keywords such as intelligent analysis of food quality and safety. The search results were further screened according to the team’s research expertise and the thematic requirements of each chapter to select the relevant references. The time frame primarily covers journal papers published between 2025 and 2026, while also appropriately citing earlier journal publications.

## 2. Data-Driven Research Paradigm for Food Quality and Safety

### 2.1. Single-Modal Data-Driven Paradigm

#### 2.1.1. Data Processing Approaches Based on Single-Device Platforms

**Figure 2:**
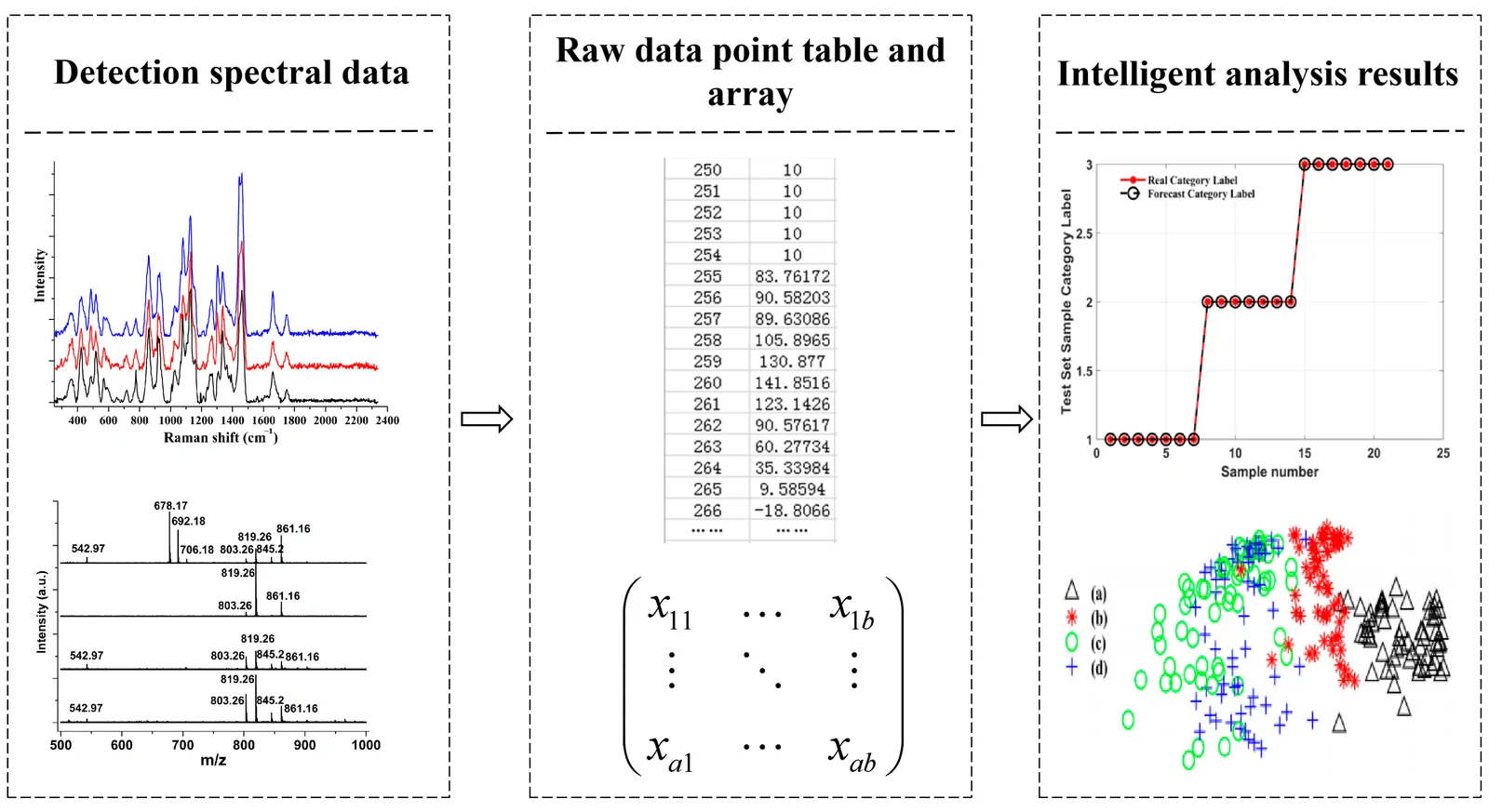
Schematic diagram of the transformation of detection data from spectra to numerical vectors (compiled from papers published by our research group) [19,20].

In food quality and safety analysis, a wide variety of detection instruments are involved, such as spectrometers, chromatographs, and mass spectrometers. However, from a data perspective, each spectrum can be represented as a vector of data, as shown in Figure 2. For Raman spectroscopy data, the horizontal axis represents wavenumber and the vertical axis represents intensity; for chromatographic data, the horizontal axis represents retention time and the vertical axis represents intensity; for mass spectrometric data, the horizontal axis represents mass-to-charge ratio and the vertical axis represents intensity. It can be observed that although the horizontal axes of data corresponding to different modal detection instruments vary, the vertical axis consistently represents intensity values, reflecting the fluctuations in intensity at different horizontal-axis positions. The peak intensity, peak width, peak shape, and ratios between spectral peaks at specific peak positions can all serve as potential feature inputs for data analysis [18,19].

When the data measured by detection instruments are combined with appropriate intelligent learning algorithms, specific food quality or safety determination tasks can be accomplished. For instance, Wang et al. proposed an improved network model, AE-YOLOv13-S, based on YOLOv13, to achieve efficient and accurate classification and detection of foreign objects such as bamboo leaf fragments, small twigs, and small pebbles that may be present in Pu-erh ripe tea [21]. Liang et al. employed hyperspectral data collected from Fragrant Pears, combined with an enhanced one-dimensional convolutional neural network (1D-CNN), to achieve efficient geographical origin identification of Fragrant Pears [22]. As summarized in Table 1, research cases on food quality or safety detection based on different single-modal data are compiled. It can be observed that although the types of data modalities are diverse, they can all be integrated with relevant intelligent algorithms to achieve prediction of characteristic component contents or quality discrimination. Meanwhile, each data modality has its own characteristics. For example, hyperspectral technology is primarily sensitive to molecules containing specific chemical bonds (such as O–H, N–H, and C–H), making it particularly suitable for characterizing key components in food, including moisture, fat, protein, and carbohydrates [23]. Raman spectroscopy, on the other hand, is especially adept at capturing vibrational information of molecular skeletons and is therefore highly sensitive to non-polar bonds (such as C–C and C=C) and molecules with symmetric structures, making it suitable for characterizing molecules such as fats, proteins, and carbohydrates [24]. If SERS technology is employed, signals of specific components, such as nitrogen-containing adulterants, can be amplified by design [25]. The technical parameters of the acquisition equipment can also affect the recognition performance. For example, Zhang et al. found that different laser integration times during Raman spectral acquisition of dairy product signals led to differences in recognition results [26]. Lintvedt et al. comparatively analyzed the robustness of models based on Raman spectroscopy and near-infrared spectroscopy through two case studies: the transferability of sugar content models for intact strawberry fruits across different seasons, and the transferability of fat content models across different degrees of meat mincing. They found that Raman spectroscopy can generate well-separated characteristic peaks related to analytes, thus offering inherent robustness advantages in chemometrics, enabling the construction of regression models with lower complexity and minimizing the risk of incorporating irrelevant and interfering spectral variations into the models. However, they also noted that prediction errors may occur when fluorescent substances interfere [27].

#### 2.1.2. Data Processing Paradigms Based on Single-Modal Two-Dimensional Correlation Spectroscopy

When a single data modality is combined with intelligent algorithms, data processing itself is also performed, such as the construction of two-dimensional correlation spectroscopy. This is a data ensemble processing approach under perturbation conditions. The basic idea is to introduce an external perturbation to the food system, then collect multiple spectral data over time, and subsequently obtain dynamic high-dimensional spectra describing the perturbation-mediated changes through two-dimensional correlation analysis [28]. Two-dimensional correlation spectra can comprehensively utilize multiple dynamic spectral data, and are expected to reveal the variation patterns of subtle but critical spectral features such as overlapping peaks, weak peaks, and shifted peaks that are often overlooked in conventional data. Meanwhile, they can also serve as data inputs combined with intelligent learning algorithms for food quality and safety analysis [29]. For example, Lafi et al. proposed a two-trace two-dimensional correlation spectroscopy (2T2D-COS) method for melamine-doped milk powder, achieving quantitative identification of adulterants [30]. Zhang et al. proposed a method for constructing laser-perturbed two-dimensional correlation Raman spectroscopy of milk powder, and combined it with similarity algorithms to achieve quantitative identification of different brands of milk powder [31]. Lapcharoensuk et al. converted one-dimensional near-infrared spectra into two-dimensional maps to enhance the feature learning capability of deep learning models. Their study found that two-dimensional continuous wavelet transform combined with the ResNet model outperformed partial least squares regression (PLSR) and other deep learning models in coconut milk adulteration detection [32]. However, this type of high-dimensional spectroscopy requires the comprehensive utilization of multiple dynamic sequential spectra, and the sampling time is somewhat increased compared with traditional spectroscopy. How to efficiently construct high-dimensional spectroscopic methods suitable for food quality and safety applications remains a research direction that requires future development.

#### 2.1.3. Single-Modal Data Preprocessing Approaches

In addition, when data from different modalities are combined with intelligent algorithms, data preprocessing is often required, such as normalization, denoising, and feature extraction, to improve recognition accuracy. Of course, some algorithms, such as CNNs, inherently contain feature extraction operations [33]. For example, Zhang et al. found that normalization processing significantly affects the recognition performance of Raman spectroscopy combined with SVM algorithms [34]. Prity proposed a novel hybrid feature extraction algorithm, GreyTexFWave, which integrates gray-level co-occurrence matrix (GLCM), gray-level dependence matrix (GLDM), texture features, fast Fourier transform (FFT), and discrete wavelet transform (DWT) features to simultaneously capture spatial-domain and frequency-domain features of rice leaf images, thereby enabling early detection of rice diseases. The recognition accuracy based on the hybrid feature extraction approach was superior to that of single feature extraction methods [35]. Li et al. found that different algorithms exhibit varying feature responses to Raman spectra, and that effective matching of appropriate features with machine learning algorithms is beneficial for improving recognition rates [36]. Moorthy et al. proposed a Residual Network-50 (ResNet-50) architecture integrating Squeeze Excitation (SE) and Cascaded Atrous Convolution (CAC), denoted as ResNet-50_SECAC. By integrating the squeeze-excitation attention mechanism and cascaded atrous convolution, the model is capable of capturing features at multiple scales while maintaining inter-channel dependencies. The squeeze-excitation mechanism enhances feature extraction capability, thereby enabling efficient classification of potato leaf diseases [37].

#### 2.1.4. Characteristics and Trends of Single-Modal Data-Driven Research

As demonstrated in numerous studies, integrating single-modal data with intelligent algorithms enables rapid and efficient analysis of food quality and safety. Nevertheless, we observe that in developing quality and safety assessment methods for specific targets, careful consideration must be given to the characteristics of the chosen data modality, particularly its responsiveness to target molecules and the state of the research subjects, as well as its overall suitability for food matrix characterization. This often entails the development of corresponding characterization techniques, including the design of SERS substrates, food sample preparation protocols, and component extraction methodologies [38]. Furthermore, when conducting data-driven food quality and safety analysis, it is necessary to comprehensively consider the applicability of machine learning algorithms, as well as various data preprocessing methods and data modality transformations [39]. Among these, effective feature extraction is particularly critical. How to extract key features from complex and diverse detection data will remain an important research topic for the foreseeable future.

**Table 1:** Food quality and safety analysis based on different single-modal data combined with machine learning algorithms.

Data Modality	Food Matrix	Core Algorithm	Research Content	Category	References
Image data	Pu-erh ripe tea	AE-YOLOv13-S (deep learning network)	Detection of foreign matter	Safety analysis	[21]
Liquid chromatography–high-resolution mass spectrometry	Pork and aquatic products	Artificial neural network	Screening and identification of unknown chemical contaminants	Safety analysis	[40]
Surface-enhanced Raman spectroscopy	Shrimp	Principal component analysis	Detection of food pollutants	Safety analysis	[41]
Surface-enhanced Raman spectroscopy	Pepper	Principal component analysis and hierarchical clustering analysis	Detection of pesticide residues	Safety analysis	[42]
Gold nanoparticle-based colorimetric method	Meat, seafood, and dairy products	Multilayer perceptron	Detection of microbial transglutaminase	Safety analysis	[43]
Hyperspectral	Fragrant Pears	Enhanced one-dimensional convolutional neural network	Identification of geographical origin	Quality analysis	[22]
Matrix-assisted laser desorption/ionization time-of-flight mass spectrometry	Milk products	Support vector machine	Discrimination of organic and conventional milk	Quality analysis	[44]
Hydrophilic interaction chromatography-charged aerosol detection	American ginseng (Panax quinquefolius)	Linear discriminant analysis	Identification of geographical origin	Quality analysis	[45]
Hyperspectral	Wheat	Random forest regression	Determination of wet gluten content	Quality analysis	[46]
Raman spectroscopy	Cymbopogon citratus essential oil	Extreme gradient boosting classification/support vector machine regression	Authentication and adulteration analysis	Quality analysis	[47]
Raman spectroscopy	Sweet potato starch and vermicelli	Meta-learning	Detection of adulteration	Quality analysis	[48]

### 2.2. Multimodal Data-Driven Paradigm

#### 2.2.1. Data Processing Approaches Based on Multiple Instruments

Multimodal data involves the comprehensive use of data collected from multiple different instruments as inputs, combined with intelligent learning algorithms to conduct food quality and safety research. For example, Wang et al. developed a zinc-air battery sensing platform and introduced a machine learning-assisted multimodal fusion strategy to achieve intelligent fusion of orthogonal electrochemical signals and photothermal signals generated by the platform. The results revealed that the performance after multimodal fusion was significantly superior to that based on prediction results from any single signal [49]. Chen et al. constructed a near-infrared and Raman multimodal spectral fusion method for non-invasive prediction of texture properties (springiness, chewiness, and hardness) of large yellow croaker fillets with different freshness levels. The results showed that spectral data after feature fusion significantly improved the performance of the PLSR model [50]. As summarized in Table 2, research cases on food quality or safety detection based on different multimodal data are compiled. It can be observed that different modal data have their own characterization characteristics, and the comprehensive utilization of the complementary characterization capabilities among modalities is expected to improve recognition accuracy. However, there also exist practical challenges, such as longer sampling time and increased computational load for recognition operations as the number of modalities increases. In addition, the same instrument may also generate data of different modalities, such as the positive and negative ionization modes of ion mobility spectrometry (IMS), and the visible and near-infrared spectral modes contained in hyperspectral imaging. Sha et al. utilized the positive and negative ionization modes of electrospray IMS to acquire spectral data of apple essence under two states, and further combined with clustering algorithms to achieve quality assessment of the samples [51].

#### 2.2.2. Multimodal Data Fusion Processing Approaches

In the process of multimodal data processing, an important issue is how to fuse the data. Common approaches include data-level fusion, feature-level fusion, and decision-level fusion [52,53]. Data-level fusion is defined as the integration of raw or minimally processed data from multiple modalities at the input layer to construct a unified input representation. In the context of multimodal data fusion, this approach typically involves the direct concatenation of multimodal data, followed by the application of intelligent learning algorithms for downstream computational tasks. Although data-level fusion is simple to implement, raw data often contain redundant or noisy signals that, if not removed, can degrade recognition accuracy. Feature-level fusion is defined as a strategy wherein features are initially extracted independently from each data modality. These high-dimensional and semantically enriched features are subsequently aligned and concatenated to construct a comprehensive feature vector, which is then input into a model for inference. The feature extraction techniques employed in this process can be categorized into mathematical operations and manual extraction. Mathematical approaches include methods such as principal component analysis (PCA), whereas manual extraction relies predominantly on the prior knowledge of domain experts for informed feature selection. For example, Tian et al. proposed a method based on near-infrared spectroscopy and Raman spectroscopy for evaluating the total volatile basic nitrogen content in Pacific white shrimp (*Litopenaeus vannamei*). The prediction model was constructed using three machine learning methods (CNNs, extreme learning machines, and backpropagation networks) combined with both data-level fusion and feature-level fusion strategies. The results showed that the feature-level fusion model achieved the best performance [54]. Feature-level fusion effectively utilizes the useful information from raw data, improving computational efficiency and recognition accuracy; however, it also faces limitations such as relatively limited feature interpretability. Decision-level fusion is defined as a strategy wherein models are trained independently for each modality, or predictions are generated separately, and the resulting decisions—such as classification outputs—are subsequently integrated using mechanisms including voting or weighted averaging. In other words, each sensor or data source first renders an independent preliminary judgment based on its own information. A fusion center then collects these individual conclusions, synthesizes them according to predefined rules and assigned weights, and ultimately derives a globally optimal decision. This strategy offers high flexibility and strong fault tolerance, but also faces challenges such as the risk of information loss and complex decision rule design [55].

The selection among these three fusion strategies involves fundamental trade-offs. Data-level fusion preserves the most complete raw information but is highly susceptible to noise and misalignment across modalities, and may lead to the curse of dimensionality when the input data are high-dimensional. Feature-level fusion offers a more flexible compromise by enabling modality-specific preprocessing and dimensionality reduction, but its performance critically depends on the quality and relevance of the extracted features, which often require expert knowledge or heuristic tuning. Decision-level fusion provides the greatest modularity and fault tolerance—if one modality fails, the others can still contribute—but at the cost of losing cross-modal interactions that could be captured by joint modeling. Consequently, there is no universally optimal fusion strategy; the choice should be guided by the specific data characteristics, application constraints, and the degree of interpretability required.

**Table 2:** Food quality and safety analysis based on different multimodal data combined with machine learning algorithms.

Data Modality	Food Matrix	Core Algorithm	Research Content	Category	References
Dual-modal biosensing platform	maize, peanuts, peanut oil	Gradient boosting	Detection of aflatoxin B1	Safety analysis	[49]
Dual-modal Ti3C2 MXene biosensor	Soybean, peanut and corn oil, soybean meal and peanut meal	Lightweight feed-forward neural network	Detection of zearalenone	Safety analysis	[56]
Near-infrared and Raman spectroscopy	Peanuts	Convolutional neural network	Detection of aflatoxin B1	Safety analysis	[57]
Colorimetric and electronic nose sensor arrays	Allium tuberosum	Convolutional neural networks and long short-term memory network attention fusion network (CLAFNet)	Detection of procymidone residue	Safety analysis	[58]
Colorimetric and electrochemical signals	Pork and milk	Artificial neural network	Detection of *Salmonella Typhimurium*	Safety analysis	[59]
Near-infrared and Raman spectroscopy	Large yellow croaker (*Larimichthys crocea*)	Partial least squares regression	Assessing freshness via textural property detection	Quality analysis	[42]
Multi-dimensional sensor data	Baijiu	Multilayer perceptron	Recognition of the fermentation stage	Quality analysis	[60]
X-ray fluorescence, Fourier-transform infrared and near-infrared spectroscopy	Black tea	Decision-level fusion using a Bayesian consensus mechanism	Food authenticity testing	Quality analysis	[61]
Electronic nose and hyperspectral	Millet and wolfberry (goji berry)	Dual-modal multi-scale feature extraction and collaborative fusion model	Identification of geographical origin	Quality analysis	[62]
Attenuated total reflection Fourier transform infrared and Fourier transform Raman spectroscopy	Honey	Partial least squares discriminant analysis	Differentiation of honey variety and harvesting year	Quality analysis	[63]
Raman and infrared spectroscopy	Camel milk powder	Gradient Boosting, Random Forest	Detection of milk powder adulteration	Quality analysis	[64]

Note: The multi-dimensional sensor data include online sensor data (temperature, CO_2_, pH, humidity), offline physicochemical data (starch, moisture, alcohol), and gas chromatography-based flavor compound data [60].

## 3. Data–Mechanism Hybrid-Driven Research Paradigm for Food Quality and Safety

### 3.1. Research Paradigm of Data–Mechanism Hybrid-Driven Approach

While effective, the purely data-driven paradigm encounters fundamental limitations that stem from its reliance on statistical associations rather than causal mechanisms. These challenges have motivated the exploration of alternative paradigms that integrate domain knowledge into the learning framework [65]. In recent years, data-free or experience-driven approaches, as well as data–mechanism hybrid-driven approaches for food quality and safety research, have also undergone rapid development [66,67]. The data–mechanism hybrid paradigm refers to the synergistic integration of data-driven approaches with mechanistic models, aiming to improve model performance with respect to predictive accuracy, interpretability, generalization, and data efficiency. Data-driven methods encompass machine learning, deep learning, and their variants, whereas mechanistic models are formulated on the basis of mathematical equations or rules grounded in prior physical, chemical, biological, or other domain knowledge. Based on the current literature, the data–mechanism hybrid-driven paradigm can be broadly classified into two primary categories: direct integration, exemplified by the physics-informed neural network (PINN); and indirect integration, typically implemented as a parallel framework combining data-driven prediction with mechanistic analysis [68].

We begin with an introduction to the PINN, which represents a new class of machine learning paradigms that integrates physical laws, typically formulated as partial differential equations, as prior knowledge directly into the training procedure of neural networks. The core principle of this research approach lies in the fact that it no longer relies solely on pure data driving. Instead, it constructs a composite loss function that includes both a data-fitting term required for traditional supervised learning (measuring the deviation between predicted values and actual observations) and a physics-constraint term (often referred to as the residual term, which measures whether the neural network output satisfies the governing equations and initial/boundary conditions at spatiotemporal sampling points). During the training process, the optimizer simultaneously minimizes both loss terms, thereby constraining the solution space of the network to a manifold that conforms to physical laws. This enables PINNs to make effective inferences even in data-sparse regions, by leveraging physical equations to guide the learning process. The result is a continuous, differentiable surrogate model that satisfies conservation laws. This paradigm is particularly effective for addressing complex forward problems like process simulation and inverse problems like the inference of unknown physical parameters in food engineering [69].

In addition, the parallel approach that combines data-driven prediction with mechanistic analysis also represents an important research pathway. Indirect hybrid models that integrate mechanisms such as mass transfer, heat transfer, reaction kinetics, microbial growth models, or chemical equilibrium constraints have drawn increasing attention from researchers [70]. For example, El-Mesery et al. employed a purely data-driven artificial neural network (ANN) model to achieve high-precision predictions of energy consumption and thermal efficiency. Meanwhile, they conceptualized the drying process as a physical phenomenon and applied classical heat and mass transfer laws (Fick’s second law) along with mathematical models (the Midilli et al. model) to describe it, thereby calculating key physical parameters such as effective moisture diffusivity and activation energy to investigate the heat and mass transfer mechanisms during the drying process [71].

### 3.2. Characteristics and Trends of Data–Mechanism Hybrid-Driven Research with Case Examples

For example, Xie et al. developed a PINN framework that uses sparse unmanned aerial vehicle (UAV) thermal infrared sensor measurements as supervised constraints, while incorporating crop phenotypic information from UAV visible-light imagery and continuous meteorological variables as inputs. The surface energy balance equation was embedded into the long short-term memory (LSTM)-Informer architecture, enabling the network to capture both local temporal dependencies and global diurnal variation patterns while adhering to the principle of energy conservation, ultimately achieving continuous hourly reconstruction and 48-h prediction of rice canopy temperature (Tc) [72]. Ma et al. evaluated the application of PINN for food kinetic modeling in cases including seed drying, bread baking, and kiwifruit softening. By integrating kinetic mechanisms into the neural network framework, the results showed that the constructed PINN models outperformed empirical models in both extrapolation and interpolation tasks [73]. Garakani et al. integrated four classical Hermia fouling mechanisms into a physics-constrained data-driven model, enabling the identification of stage transitions among dominant fouling mechanisms and the quantification of their relative contributions. This model exhibited both high predictive accuracy and low uncertainty, outperforming classical fouling models while also offering mechanistic interpretability [74]. Hu et al. proposed a PINN for food production control, which could accurately predict indoor climate and crop status in controlled environments based on control inputs (including temperature, humidity, CO_2_, irrigation, and fertilization control). The method was applied to tomato cultivation environment control and achieved satisfactory results [75]. As a novel research paradigm, its advantages over purely data-driven approaches lie in its superior interpretability, extrapolation and generalization capabilities, and data efficiency. Purely data-driven models are akin to “black boxes” and, in the highly variable and non-stationary environment of the food supply chain, are highly susceptible to prediction failures due to data distribution shifts, while also making it difficult to trace the root causes of risks. In contrast, the mechanism-driven paradigm embeds established physical, chemical, and biological laws from food science into model training, ensuring that the predictions inherently conform to scientific principles and remain logically sound even under data-sparse or extreme conditions. Moreover, it can intuitively explain the physical or biological causes of risks through internal model parameters, providing actionable decision-making bases for precise regulation and traceability interventions, thereby constructing an intelligent food safety assurance system that is both accurate and trustworthy. Nevertheless, hybrid models generally entail greater training complexity compared to purely data-driven approaches, and the appropriate formulation of physical constraints requires meticulous design. In addition, their predictive performance is not guaranteed to consistently exceed that of well-established data-driven baselines. As shown by Walter et al., the performance discrepancy between purely data-driven ANN and PINN diminishes substantially when observational data are sufficiently dense, whereas the advantages of PINN become evident primarily under data-sparse conditions. This suggests that, given abundant high-quality labeled data, purely data-driven models may achieve comparable performance and offer greater ease of deployment for certain applications [76]. Furthermore, we observe that the application of this paradigm in the food quality and safety domain remains limited in the current literature. This scarcity may stem from the inherent complexity of food systems, which presents theoretical challenges in formulating precise mathematical and physical descriptions of their underlying mechanisms. Moreover, given that the data–mechanism hybrid paradigm is still in its nascent stage, concerted research efforts are urgently needed to develop more robust and reliable case studies in this field.

Examining the above case studies collectively reveals several patterns. First, PINNs demonstrate the most significant advantages over purely data-driven approaches in scenarios where underlying physical mechanisms are well-characterized but data are inherently sparse, such as in thermal process modeling and drying kinetics. In these cases, the physical constraints effectively compensate for the scarcity of labeled data by enforcing physically plausible solutions. Second, the manner in which physical knowledge is embedded varies across applications: while Xie et al. [72] and Hu et al. [75] incorporated energy balance equations as soft constraints in the loss function, Ma et al. [73] integrated kinetic rate laws directly into the network architecture. This suggests that the optimal integration strategy is context-dependent and requires case-specific tailoring. Third, a common challenge emerging from all reported studies is the difficulty in balancing the data-fitting and physics-constraint terms during training, which often requires extensive hyperparameter tuning and may limit the practical accessibility of PINN for non-specialist users. These observations highlight that while the data–mechanism hybrid paradigm holds great promise, its effective application demands both domain expertise in food science and technical proficiency in scientific machine learning.

## 4. Conclusions and Future Outlook

Traditionally, the data-driven paradigm has been the dominant approach in food quality and safety research. By acquiring single-modal or multimodal data from food objects and combining them with machine learning algorithms, this paradigm directly mines complex implicit correlations and nonlinear patterns from massive, high-dimensional, multi-source, and heterogeneous detection data. Particularly when dealing with food systems whose underlying mechanisms are not yet fully understood, it demonstrates strong modeling flexibility and predictive accuracy, and is capable of capturing subtle fluctuations and combined risks that are easily overlooked by traditional detection methods, enabling earlier and more sensitive warnings, and facilitating food safety assessment or quality control evaluation. However, as research has progressed, the purely data-driven paradigm has also revealed some shortcomings, such as: (1) poor model interpretability, especially in the case of deep learning models, where decision-making processes are difficult to understand intuitively; (2) concerns regarding extrapolation and generalization capabilities—purely data-driven models essentially learn the statistical distribution of training data, and once the environment changes, data distribution shifts can lead to sharp declines in model performance; and (3) extreme dependence on high-quality data—model performance is severely constrained by the quantity, quality, and representativeness of training data. In the food domain, issues such as data silos, high annotation costs, and severe imbalance between positive and negative samples are prevalent, which readily lead to model overfitting or learning spurious correlations. To overcome these problems, researchers have begun to explore a novel research paradigm that integrates data with mechanisms, preserving the advantages of data fitting while incorporating prior knowledge from physics and biology as constraints and guidance for the model, thereby achieving both accurate and reliable intelligent food safety management and control.

Notwithstanding the conceptual appeal of the data–mechanism hybrid paradigm discussed above, its transition from theory to practice is confronted with a number of tangible challenges that call for concerted research efforts. At the data level, bottlenecks persist. Food systems are inherently dynamic and complex, with raw material batch-to-batch variability, fluctuations in processing parameters, and alterations in storage and logistics conditions frequently giving rise to domain shifts. As a result, models that demonstrate high accuracy in controlled laboratory settings may exhibit substantial performance degradation when deployed on actual production lines. In addition, the procurement of high-quality annotated data is both labor-intensive and cost-prohibitive, relying heavily on specialized equipment and expert annotation. The limited interoperability of data across diverse food matrices further compounds the scarcity issue. At the model level, transferability and standardization emerge as major obstacles. The majority of existing models are custom-built for specific food matrices and particular instrument platforms, and the lack of standardized calibration protocols and evaluation benchmarks impedes cross-platform reusability and large-scale industrial adoption. At the computational level, the balance between resource demand and real-time operability presents a nontrivial challenge. Hybrid architectures, especially PINN and related physics-constrained frameworks, entail substantial computational overhead due to the repeated evaluation of partial differential equation (PDE) residuals and automatic differentiation, which may preclude their deployment on edge devices or in time-sensitive online monitoring scenarios. Furthermore, the paucity of external validation remains a pervasive gap in the literature. Most reported studies are confined to proof-of-concept demonstrations at the laboratory scale, with limited evidence of systematic blind testing or cross-validation under realistic production conditions involving multiple devices and batch variations. The practical robustness and reliability of the proposed models therefore remain to be rigorously established. To address these challenges, future investigations should prioritize the following avenues: (1) advancing few-shot learning, transfer learning, and domain adaptation techniques to bolster model generalization in data-scarce and cross-domain contexts; (2) constructing publicly available benchmark datasets and standardized evaluation protocols tailored to food quality and safety, thereby enabling fair comparisons and accelerating methodological innovation; (3) designing lightweight network architectures and computationally efficient training regimes to accommodate real-time deployment constraints; and (4) fostering interdisciplinary partnerships to conduct field studies that apply hybrid modeling approaches to a wider spectrum of food processing, logistics, and microbial risk assessment scenarios, thereby generating practical deployment insights. Ultimately, a virtuous interplay between theoretical innovation and engineering practice will be essential to realize the transition of intelligent food quality and safety analytics from the laboratory to industrial reality.

## Data Availability

No new data were created or analyzed in this study. Data sharing is not applicable to this article.
